# Not a Stroke, but a Virus: When a Slurred Call Tells a Different Story

**DOI:** 10.7759/cureus.98087

**Published:** 2025-11-29

**Authors:** Inês C Teixeira, Filipa Andrade, Sara Gouveia, Marta A Caldeira

**Affiliations:** 1 Family Medicine, Centro de Saúde do Bom Jesus, ACES Madeira SESARAM, Funchal, PRT; 2 Family Medicine, Centro de Saúde da Ribeira Brava, ACES Madeira SESARAM, Ribeira Brava, PRT; 3 Family Medicine, Centro de Saúde de Boaventura, ACES Madeira SESARAM, São Vicente, PRT

**Keywords:** neuropathic pain, peripheral facial paralysis, postherpetic neuralgia, ramsay hunt syndrome, varicella-zoster virus

## Abstract

A skin rash in the auricular and cervical regions, accompanied by severe pain, was identified as an atypical presentation of Ramsay Hunt syndrome (RHS). This condition, resulting from the reactivation of the varicella-zoster virus (VZV) in the geniculate ganglion, is characterized by peripheral facial paralysis, auditory disturbances, and, in some cases, unusual cutaneous manifestations that can complicate diagnosis. We report the case of a 71-year-old male with a history of cardiovascular disease, who presented with insidious onset of dysarthria and right peripheral facial paralysis over the course of 10 days, along with vesiculopustular lesions in the C2-C3 dermatomes, complicated by bacterial superinfection and moderate-to-severe sensorineural hearing loss. A combined treatment regimen of antiviral, antibiotic, and corticosteroid therapy was initiated, followed by an intensive multidisciplinary rehabilitation plan. After 65 days, he continued to experience severe motor deficits and chronic neuropathic pain, despite physiotherapy and gradual optimization of gabapentinoid therapy.

This report highlights how atypical presentations and delayed diagnosis can adversely affect prognosis, emphasizing the importance of early antiviral and corticosteroid intervention to minimize motor, auditory, and pain-related sequelae. It also underscores the importance of integration between primary and hospital care to ensure continued rehabilitation, symptomatic control, and preservation of quality of life in elderly patients with comorbidities.

## Introduction

Ramsay Hunt syndrome (RHS) results from the reactivation of the varicella-zoster virus (VZV), the agent responsible for Chickenpox in childhood, which remains latent in nerve ganglia and may later reactivate in the form of herpes zoster. In RHS, this reactivation affects the geniculate ganglion of the facial nerve, causing peripheral facial paralysis with cutaneous involvement and, frequently, auditory symptoms [1]. It represents an uncommon complication of VZV, with an estimated incidence of five cases per 100,000 people per year, accounting for approximately 7% of non-traumatic peripheral facial paralyses. Although it can affect individuals of any age, it is more prevalent in individuals aged 70-89 and in those with compromised immune systems. Advancing age alone is an important risk factor for more severe or atypical presentations [1,2].

The classic clinical presentation of RHS consists of the triad of ipsilateral facial paralysis, otalgia, and vesicular eruption in the external auditory canal, auricle, or oral mucosa. However, atypical forms have also been reported, including involvement of cervical dermatomes, absence of rash - “zoster sine herpete” - or the presence of secondary bacterial infections, all of which may complicate diagnosis and delay treatment initiation [3,4]. Cervical involvement may result from the spread of reactivated VZV along sensory pathways beyond the geniculate ganglion, as the virus can remain latent in multiple cranial and dorsal root ganglia and reactivate in more than one segment [1,3].

Although uncommon, atypical presentations of RHS carry important clinical implications, as they often lead to delayed diagnosis, suboptimal therapeutic windows, and increased risk of persistent neurological deficits. Their low prevalence and diagnostic ambiguity underscore the relevance of reporting such cases to improve recognition and management [5,6]. Involvement of the vestibulocochlear nerve (cranial nerve VIII) is possible and may lead to hearing loss, vertigo, and tinnitus, which are often irreversible and sometimes difficult to manage [7].

Prognosis is strongly related to the promptness of diagnosis and the initiation of antiviral and corticosteroid therapy, which is most effective when started within the first 72 hours. While benefits have been observed even beyond this window, treatment initiated after seven days is progressively less effective, often resulting in incomplete recovery of facial function [2]. Additionally, complications such as chronic neuropathic pain with allodynia are common, especially in older patients or those with comorbidities, necessitating a prolonged, multidisciplinary management approach [8].

## Case presentation

The patient was a 71-year-old male, autonomous, with a medical history of arterial hypertension, dyslipidemia, cerebrovascular disease with an ischemic stroke in 2013 without sequelae, obesity, and depressive disorder. His current medications included olmesartan 40 mg + hydrochlorothiazide 25 mg daily, lercanidipine 20 mg daily, nebivolol 5 mg daily, atorvastatin 20 mg daily, clopidogrel 75 mg daily, sertraline 50 mg daily, and lorazepam 1 mg as needed. He had no known drug allergies and was up to date with the National Vaccination Program. He presented to the peripheral urgent care service with complaints of slurred speech of sudden onset, with approximately four hours of onset, noted by his daughter during a phone call. He also reported skin lesions in the right auricular and cervical region, accompanied by intense pain, with a 10-day history, initially attributed to a shaving blade cut. He described progressive worsening of the lesions, retroauricular pain, chills, and general malaise.

**Figure 1:**
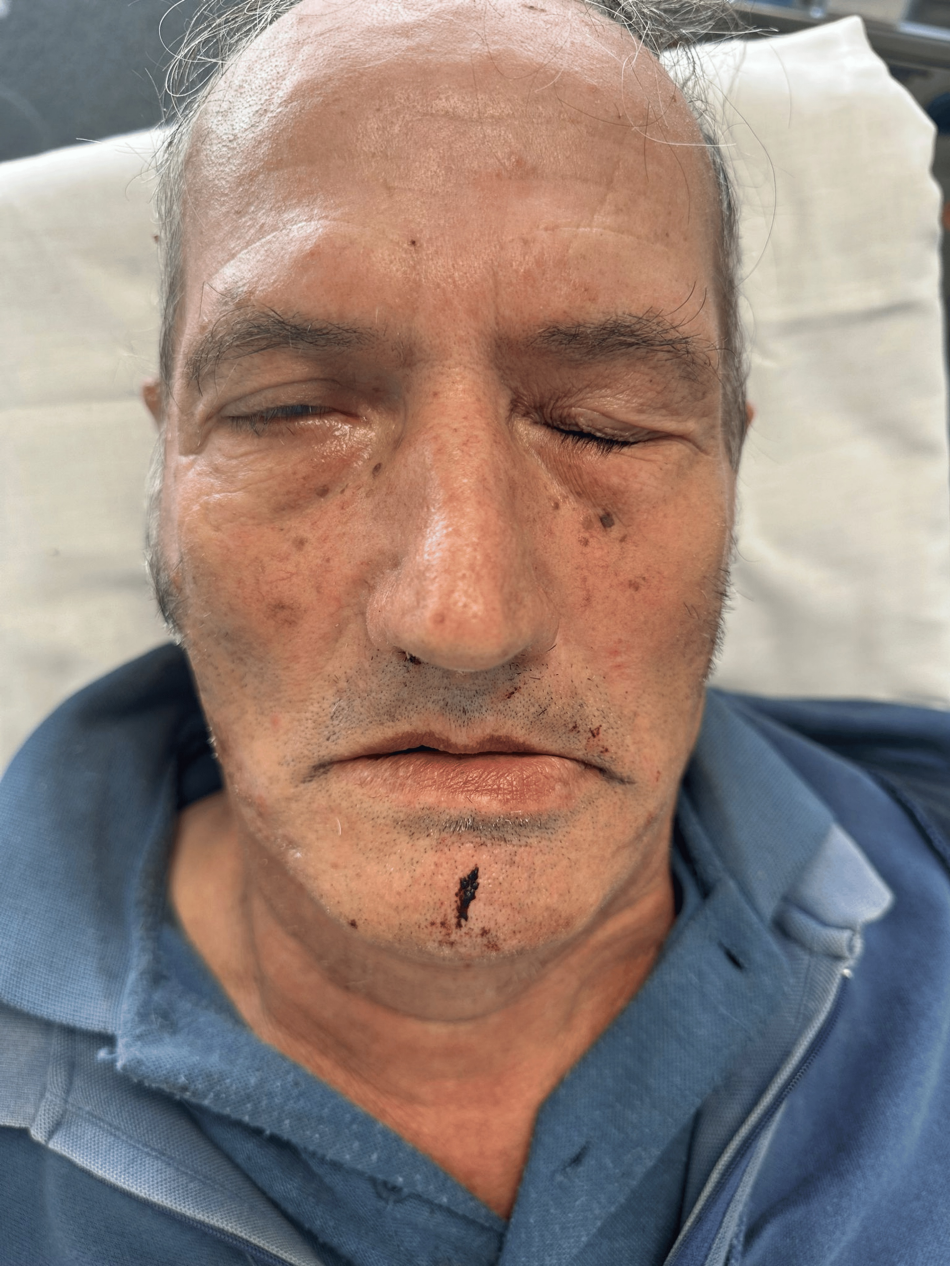
Right-sided facial paralysis with inability to close the eye

**Figure 2:**
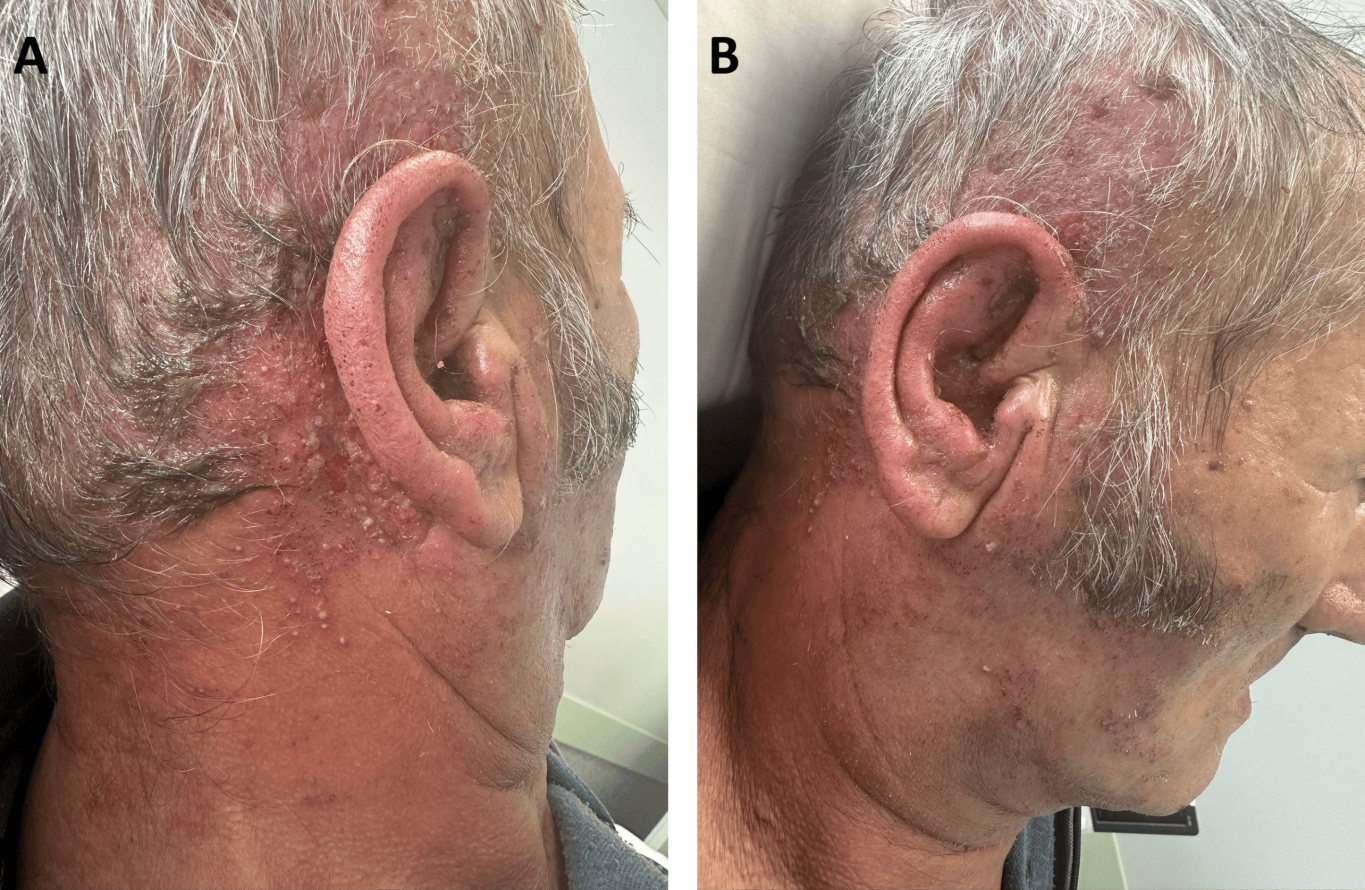
Vesiculopustular lesions on the scalp, retroauricular region (A), and in the external auditory canal (B)

On examination, the patient was conscious and cooperative, hemodynamically stable, with a blood pressure of 154/84 mmHg, heart rate of 80 bpm, oxygen saturation of 98% on room air, and a temperature of 36.4 ºC. Neurological examination revealed mild dysarthria and a right-sided peripheral facial palsy (Figure 1), graded as House-Brackmann V. Extensive vesiculopustular and crusted lesions were observed over the right retroauricular area, mastoid region, and upper cervical zone, extending towards the scalp (Figure 2A). The lesions were grouped along the C2-C3 dermatomes and associated with marked erythema and edema. Some vesicles had ruptured, with seropurulent exudate suggesting secondary bacterial superinfection, likely consistent with erysipelas. The external auditory canal also appeared inflamed, with erythematous and edematous skin changes (Figure 2B).

The patient was referred to the central hospital for further evaluation and clinical management, where he was assessed by the Internal Medicine Department. Following clinical and neurological evaluation, peripheral facial paralysis in the context of VZV infection was confirmed, consistent with a diagnosis of RHS. Treatment was initiated with valaciclovir 1 g every eight hours, prednisolone 30 mg/day for five days, and amoxicillin + clavulanic acid 875/125 mg every eight hours for 10 days. The patient was discharged with referral to Physical and Rehabilitation Medicine (PRM) and Otorhinolaryngology (ORL) consultations.

He was evaluated in the PRM outpatient clinic 15 days after the initial episode, initiating a nutritional supplement enriched with vitamins B1 and B12, lubricating eye drops, and a functional rehabilitation program with a physiotherapist at the local Primary Health Care Center. One week later, he returned to the Emergency Department due to severe neuralgia in the right hemiface and cervical region. Gabapentin was initiated at 300 mg/day and later increased to 500 mg/day, resulting in partial pain relief but persistent allodynia. In ORL consultation, moderate-to-severe right-sided sensorineural hearing loss was diagnosed, and a hearing aid adaptation process was initiated, which is currently ongoing. He was re-evaluated in PRM consultation 65 days after the initial episode, showing persistent right-sided facial asymmetry, incomplete eyelid closure with positive Bell’s sign, and limited residual movement of the right nasal wing and lip. The facial paralysis remained House-Brackmann grade V, with minimal clinical improvement.

**Figure 3:**
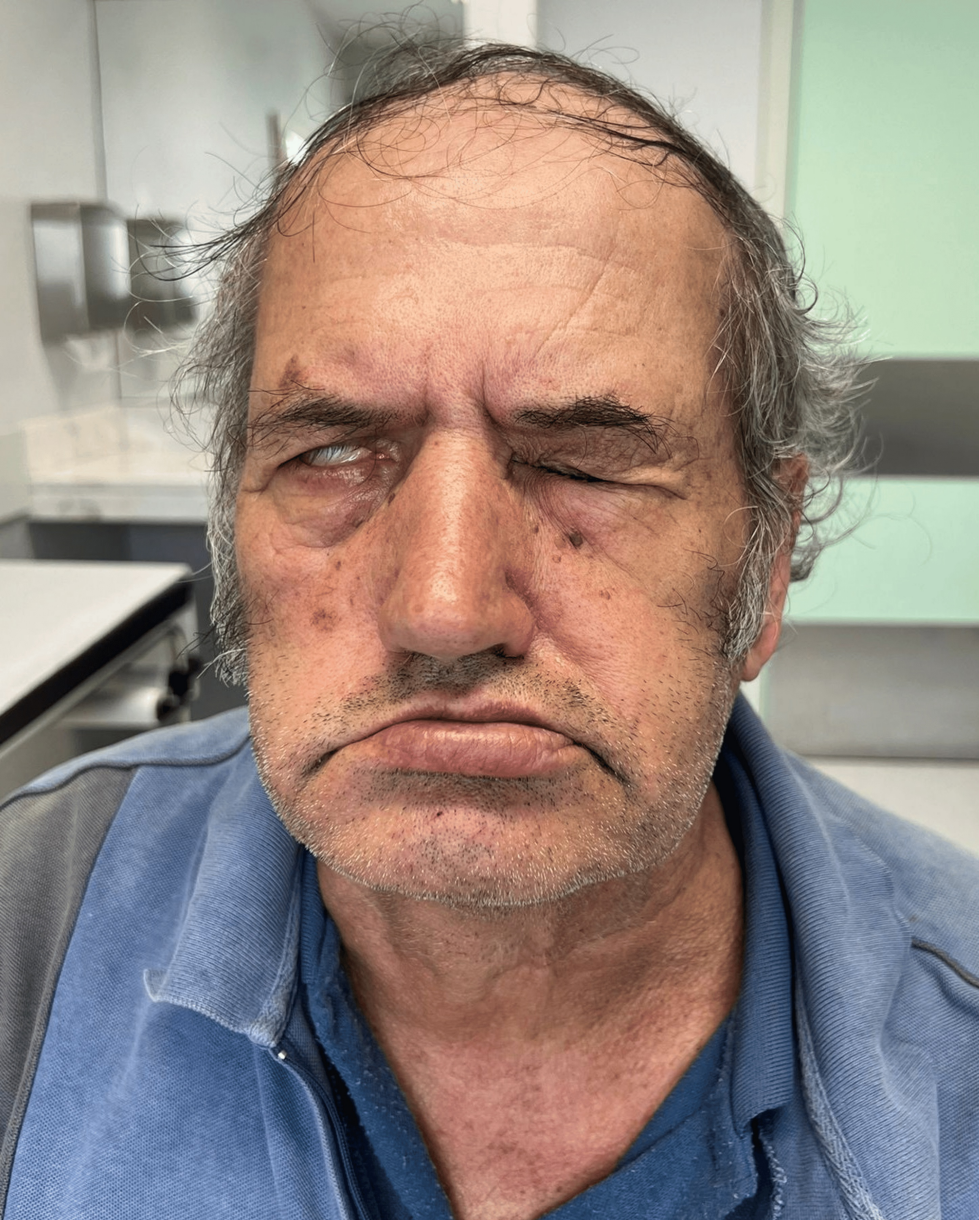
Persistent right-sided facial asymmetry with incomplete eyelid closure

The patient was enrolled in a functional rehabilitation program with a physiotherapist at his local Health Center and continued to experience neuropathic pain in the right hemiface, treated with pregabalin 300 mg/day and gabapentin 500 mg/day. During a follow-up visit with his Family Physician, the objective examination revealed unchanged functional disabilities with persistent facial paralysis (Figure 3), and due to ongoing pain complaints, pregabalin was then titrated to 400 mg twice daily, with short-term re-evaluation planned.

He was re-evaluated in the PRM consultation 121 days after the initial episode, maintaining facial asymmetry at rest, a positive Bell’s sign on the right side, and had a slight recovery with minimal voluntary movement of the right nasal wing and lip. Facial paralysis persisted at House-Brackmann grade V, showing virtually no improvement since the previous assessment. He continued to experience acute neuropathic pain, leading to the initiation of tapentadol 150 mg every 12 hours and referral to the Pain Medicine consultation.

At the Pain Medicine consultation, 124 days after the initial episode, his chronic pain medication regimen was reviewed and adjusted to duloxetine 30 mg daily for seven days, with escalation to 60 mg daily thereafter; tapentadol 150 mg every 12 hours; pregabalin 300 mg every 12 hours; magnesium metamizole 575 mg and paracetamol 1 g every eight hours as needed; and lidocaine-prilocaine 25+25 mg/mL topical cream as needed. A CT scan of the neck soft tissues and paranasal sinuses was requested for further evaluation, and a follow-up appointment was scheduled.

To date, the patient continues multidisciplinary follow-up in Physical and Rehabilitation Medicine for ongoing motor and functional rehabilitation, and Otorhinolaryngology for hearing loss management and hearing aid adaptation. He is also followed up by the Pain Medicine team for monitoring of chronic neuropathic pain and therapeutic adjustments, and by Internal Medicine as part of long-term follow-up since his ischemic stroke in 2013. Regular assessments with his Family Physician at the local Primary Health Care Center ensure coordination of care and optimization of chronic medication management.

## Discussion

RHS is a neuroinfectious condition resulting from the reactivation of VZV in cranial sensory ganglia, with the geniculate ganglion of the facial nerve being the most commonly affected. The disease may anatomically extend to the vestibulocochlear nerve, explaining the high prevalence of associated auditory and vestibular symptoms [7]. This report highlights several relevant clinical aspects: the extension of lesions to the C2-C3 cervical dermatomes, an atypical but previously documented presentation with potential to hinder initial diagnosis [3,5]; and the presence of bacterial superinfection, in this case compatible with erysipelas. Although uncommon, such superinfections may coexist with RHS and support the use of combined antibiotic therapy for more rapid and effective management [4,9].

The clinical course was marked by persistent intense neuropathic pain, with allodynia and hyperalgesia, consistent with post-herpetic neuralgia, frequently observed in elderly patients and associated with incomplete nerve function recovery [8,10]. Involvement of the VIII cranial nerve, confirmed by moderate-to-severe sensorineural hearing loss, occurs in up to 50% of cases and is associated with a poorer auditory prognosis, particularly when antiviral therapy is delayed [6,10]. The most recent evidence supports the use of antivirals (acyclovir, valacyclovir, or famciclovir) in combination with high-dose corticosteroids, ideally initiated within the first 72 hours of the clinical picture, to maximize functional recovery and reduce sequelae [1,10]. This prompt approach improves facial recovery rates and reduces the incidence of chronic pain, although it does not eliminate the risk of auditory sequelae [6,10].

In addition to pharmacological treatment, facial motor rehabilitation plays a central role in neurological recovery and is recommended at an early stage, after the acute phase of the disease, once the initial inflammatory process has stabilized. In the management of the most frequent complaints, neuropathic pain requires a personalized approach and often includes drugs such as gabapentin, pregabalin, or tricyclic antidepressants, though their effectiveness may vary [10]. From a prognostic perspective, facial motor recovery is generally better than auditory recovery. The presence of hearing loss, advanced age, and intense pain, as seen in this case, are associated with poorer outcomes and may persist over prolonged or even permanent periods [2,6], often necessitating the use of hearing aids for functional auditory rehabilitation.

## Conclusions

RHS is a severe and frequently underdiagnosed neurological condition, particularly when it presents atypically or in elderly patients with multiple comorbidities. The clinical presentation described, with cervical involvement and bacterial superinfection, highlights the importance of a prompt, multidisciplinary, and individualized medical approach. Early initiation of antivirals and corticosteroids, combined with functional rehabilitation and symptomatic pain management, can improve clinical outcomes. In this case, delayed initiation of specific therapy may have contributed to the limited recovery observed, including persistent motor deficits, hearing loss, and chronic neuropathic pain that proved difficult to manage. This report highlights the importance of early intervention, tailored rehabilitation, and continuous follow-up in minimizing the functional impact of RHS. It also demonstrates the importance of effective coordination between hospital care and primary health care in ongoing management and rehabilitation, supporting meaningful improvement in patient autonomy and well-being.
